# Simulated High Throughput Sequencing Datasets: A Crucial Tool for Validating Bioinformatic Pathogen Detection Pipelines

**DOI:** 10.3390/biology13090700

**Published:** 2024-09-06

**Authors:** Andres S. Espindola

**Affiliations:** Department of Entomology and Plant Pathology, Oklahoma State University, Stillwater, OK 74078, USA; andres.espindola@okstate.edu

**Keywords:** controls, benchmark, HTS, pathogen, detection, sequencing, simulation

## Abstract

**Simple Summary:**

Validation of plant pathogens’ diagnostic assays requires positive and negative controls. High Throughput Sequencing (HTS) has shown capabilities for detecting multiple pathogens simultaneously. However, accurate pathogen identification from HTS data depends on the bioinformatic pipeline used. There is currently no consensus on the best pipeline, leading to inconsistent results. To address this, standardized artificial HTS datasets are proposed as benchmarks to evaluate the performance of bioinformatic pipelines used in detection scenarios using HTS. These datasets will aid in resolving challenges like unknown sensitivity and specificity of the bioinformatic tool, contributing to advancing plant pathogen detection using HTS.

**Abstract:**

The validation of diagnostic assays in plant pathogen detection is a critical area of research. It requires the use of both negative and positive controls containing a known quantity of the target pathogen, which are crucial elements when calculating analytical sensitivity and specificity, among other diagnostic performance metrics. High Throughput Sequencing (HTS) is a method that allows the simultaneous detection of a theoretically unlimited number of plant pathogens. However, accurately identifying the pathogen from HTS data is directly related to the bioinformatic pipeline utilized and its effectiveness at correctly assigning reads to their associated taxa. To this day, there is no consensus about the pipeline that should be used to detect the pathogens in HTS data, and results often undergo review and scientific evaluation. It is, therefore, imperative to establish HTS resources tailored for evaluating the performance of bioinformatic pipelines utilized in plant pathogen detection. Standardized artificial HTS datasets can be used as a benchmark by allowing users to test their pipelines for various pathogen infection scenarios, some of the most prevalent being multiple infections, low titer pathogens, mutations, and new strains, among others. Having these artificial HTS datasets in the hands of HTS diagnostic assay validators can help resolve challenges encountered when implementing bioinformatics pipelines for routine pathogen detection. Offering these purely artificial HTS datasets as benchmarking tools will significantly advance research on plant pathogen detection using HTS and enable a more robust and standardized evaluation of the bioinformatic methods, thereby enhancing the field of plant pathogen detection.

## 1. Introduction

High-throughput sequencing (HTS) technologies, coupled with the reduction in sequencing costs, are revolutionizing the field of plant diagnostics by enabling rapid, cost-effective, and simultaneous pathogen detection in a single sample with promising sensitivity and high specificity [1,2]. The adoption of HTS in plant health diagnostics represents a significant advancement since the introduction of PCR-based detection in the late 1980s, offering a powerful method for comprehensive pathogen detection without prior assumptions about the organisms present [3]. HTS techniques such as amplicon sequencing (also known as metabarcoding) [4] and shotgun metagenomics have been applied across a variety of plant pathogens, including bacteria, viruses, fungi, and nematodes [5,6]. A clear example of how HTS has revolutionized plant diagnostics is shown by its ability to detect any plant virus in a sample without prior knowledge, thereby facilitating the discovery of new plant viruses [7]. HTS has been successfully applied for virus and viroid discovery in various agricultural crops, leading to its adoption in routine pathogen detection [8,9]. The application of HTS in plant pathology has allowed for the identification of viruses, bacteria, fungi, and oomycetes infecting many agricultural crops [10,11,12,13,14,15,16,17,18,19,20].

The versatility of HTS extends to the identification of specific pathogens at various taxonomic levels and groups, as demonstrated by tools like MARPLE, which enable rapid identification of individual pathogen strains directly from field-collected infected plant tissue [21]. Furthermore, portable HTS technologies like the MinION from Nanopore have opened new avenues for point-of-care detection and identification of plant pathogens, offering rapid and accurate identification of dominant pathogenic organisms from plant tissues [22,23]. These technologies have shown reproducibility and sensitivity in detecting various plant pathogens, including viruses and viroids [8,9].

Despite its advantages, the implementation of HTS in routine plant diagnostics is fraught with challenges. One major hurdle is the lack of standardized bioinformatic pipelines and standard methodological procedures when analyzing the output data [24]. Despite some government regulatory agencies beginning to offer guidelines for evaluating HTS for pathogen diagnostics in wet labs and bioinformatics pipelines, these guidelines are still quite general [25,26]. This may be due to a shortage of peer-reviewed literature recommending methods for evaluating these pipelines. This lack of consensus has been associated with the lack of trustworthy controls or HTS datasets that could serve as a benchmarking method for bioinformatic pipelines [27]. Such controls should contain known amounts of pathogens and must include all potential artifacts generated by various variables in the sequencing platform and the host–pathogen sample. To address this, researchers created a collection of 18 datasets, including semi-artificial, real, and fully artificial datasets, to test different aspects and challenges in plant virus detection from HTS data. However, the limitations of this study were that all datasets were sequenced or/and simulated using an Illumina system, with most using a four-channel system. This could be problematic when using benchmarked pipelines on datasets from two-channel systems, which are known to have erroneous guanine base calls [27]. Additionally, the use of real sequencing datasets is a great limitation when benchmarking these pipelines because the absolute amount of pathogen in the sample cannot be known with certainty [27]. 

The scientific community and stakeholders clearly require a comprehensive methodology to create HTS datasets that can be used to benchmark the bioinformatic pipelines that are used for plant health diagnostics. Even though the literature related to the use of HTS datasets for this type of benchmarking is scarce, it is important to note that having these datasets will play a pivotal role in ensuring the robustness and reliability of pathogen detection methods, ultimately contributing to better disease surveillance and management. Here, we will explore the importance of and considerations for developing mock standardized HTS datasets to benchmark bioinformatic pathogen detection pipelines, and discuss the various drawbacks and benefits of artificial datasets as benchmarking controls for bioinformatic pipelines used in plant health diagnostics. 

## 2. Types of Datasets

Two main types of reference datasets can be used: real and artificial. Real datasets derived from actual biological samples provide realistic scenarios encountered by researchers, but their use is limited because the “true” composition of the samples is not known with absolute certainty. Artificial datasets, created entirely in silico, allow for complete control over their composition, but they may not accurately reflect the complexities of real HTS data. To overcome these limitations, semi-artificial datasets, which combine real HTS data with artificially generated reads, have been used in the past [27]. Researchers should consider the impact of incorporating the complexity of the real HTS dataset on any diagnostic performance metrics within bioinformatic pipelines. Analytical sensitivity and analytical specificity are fundamental diagnostic performance metrics for any diagnostic assay. Analytical sensitivity refers to the ability of an assay to detect low concentrations of the target analyte (pathogen reads) accurately, while analytical specificity pertains to the ability of the assay to correctly identify the target analyte without interference from other substances [28,29]. Constructing a serial dilution of the pathogen is often performed with nucleic acids and water for PCR analytical sensitivity. On the other hand, to evaluate HTS diagnostic assays, we have suggested the use of serial dilutions using the pathogen and host nucleic acids as a background or using infected samples with known concentrations of the pathogen obtained either by real-time PCR [30,31]. However, the costs of library preparation and sequencing do not yet allow us to perform full experimental design for each pathogen. Therefore, artificial datasets are imperative when calculating analytical sensitivity. The most fundamental analytical sensitivity experiment uses a matrix that does not interact with the analyte (host and pathogen). However, the matrix of the sample can influence analytical sensitivity; therefore, subsequent analytical sensitivity experiments should include a more complex matrix. There may be multiple factors influencing the matrix depending on the sampling technique, host, environmental conditions, nucleic acid extraction, enrichment, and library preparation methods. Recreating all possible scenarios is not cost-efficient. Therefore, the most prevalent matrix should be used; for example, if a host–pathogen sample is often found as a co-infection or multiple infections, then the matrix must include that interaction. Therefore, at different tiers of validation, the use of artificial or real HTS datasets is interchangeable (Table 1).

Do we need to replicate the intricacies of a real sample exactly to generate exact analytical sensitivity and specificity values? If so, how do analytical sensitivity and specificity values vary when comparing real and artificial scenarios, and are these variations significant? Therefore, a hypothesis that needs to be addressed is whether the disconnect between artificial HTS datasets and real-world complexities shown in real HTS datasets can potentially lead to an overestimation or underestimation of the analytical sensitivity of an HTS test. Determining this difference in diagnostic performance metrics using available bioinformatic tools used in plant health HTS tests can be achieved as a proof of concept and should include different taxonomic groups, sampling techniques, and seasonality, as well as wet lab pipelines that could introduce the variation that is observed in real HTS datasets. Additionally, to make the diagnostic performance metric results comparable, all bioinformatic pipelines used in the evaluation should generate positive/negative results as an output, which may be challenging because many bioinformatic tools used for plant health diagnostics provide results that require researchers or diagnosticians to evaluate their output to consider the pathogen’s presence in the sample [3]. Hence, existing bioinformatic pipelines should produce impartial and objective results. These results should indicate positive or negative outcomes while still allowing for interpretation as either absolute or relative values generated by the pipeline. By addressing these gaps, researchers can determine if artificial HTS datasets are sufficient, and the assessment of bioinformatic pipelines used for HTS diagnostic tests will become more consistent and supported by scientific rigor.

## 3. Existing HTS Simulators for Benchmarking Bioinformatic Pipelines for Pathogen Detection

HTS simulators are essential for evaluating the performance of bioinformatic pipelines, especially for pathogen detection, where knowing the exact composition of a sample is crucial for accurate diagnosis. Here, we describe simulators, along with their strengths and limitations, that have shown potential for aiding in the simulation of HTS data that can ultimately be used for benchmarking bioinformatic pipelines used for pathogen detection (Table 2).

ART is a widely used simulator that can generate reads with both substitution and insertion-deletion errors, making it suitable for simulating data from various sequencing platforms like Illumina and 454 [32]. However, its limitations in simulating indels, particularly their distribution, can impact the accuracy of downstream analysis [33]. BEAR utilizes a machine-learning approach to generate reads with lengths and quality scores resembling empirically derived distributions from various sequencing platforms [32]. Its strength lies in its ability to emulate data from platforms like Ion Torrent, for which dedicated simulators are limited. BEAR also automates abundance profile generation, which is often arduous in metagenomic simulations [32]. CAMISIM is a highly modular metagenome simulator that stands out for its ability to model diverse microbial communities, including real and simulated strain-level diversity. Notably, CAMISIM can generate data from various sequencing technologies, including second- and third-generation platforms, making it suitable for benchmarking a broader range of bioinformatic pipelines. It also provides gold standards for various downstream analyses, such as assembly, binning, and taxonomic profiling, enabling comprehensive evaluation of metagenomic pipelines [34]. CuReSim is specifically designed to evaluate mapping algorithms used in HTS data analysis. Its customizable nature allows the generation of reads with varying lengths, error rates, and distributions of insertions, deletions, and substitutions, making it suitable for assessing the robustness of mapping algorithms under different error scenarios [35]. FASTQSim is a platform-independent simulator that characterizes HTS datasets and generates in silico reads with matching error profiles. It is particularly useful for evaluating metagenomic algorithms, as it allows the creation of spiked datasets with known concentrations of different organisms, enabling accurate assessment of their performance in complex mixtures [33]. Grinder is a versatile simulator capable of emulating data from various platforms, including Sanger, 454, and Illumina. While it allows the creation of multi-sample datasets, its application is limited to simulating differential abundances [36]. MetaSim focuses on simulating metagenomic data and supports the use of empirical error profiles. However, it does not generate quality values for the simulated reads, potentially limiting its utility for evaluating pipelines that rely on quality scores for analysis [37]. NeSSM is specifically designed for metagenomic simulations and incorporates realistic features like sequencing error models based on explicit error distributions and sequencing coverage bias. It provides tools to estimate these parameters directly from existing metagenomic data, enhancing the fidelity of the simulated data. NeSSM supports both 454 and Illumina platforms, and a GPU-accelerated version is available for faster simulations [38]. However, the link provided for the source code was not available when we wrote this article. MetaSPARSim is specifically designed to simulate 16S rRNA gene sequencing count data. It aims to address the lack of realistic simulation models for 16S data by capturing characteristics such as sparsity and compositionality commonly observed in real datasets [39]. ReSeq is a more recent simulator that aims to improve the realism of simulated Illumina gDNA sequencing data by incorporating features like systematic errors, a fragment-based coverage model, and sampling-matrix estimates [40]. NanoSim is specifically designed to generate realistic ONT read data and allows users to characterize and utilize their own datasets to tailor the simulations [41]. The choice of simulator depends on the specific research question, sequencing platform, and the desired level of realism in the simulated data. It is important to note that while these simulators aim to capture the characteristics of real sequencing data, they may not fully encompass the complexities and biases inherent in actual experiments. Therefore, it is crucial to carefully consider the limitations of each simulator and interpret the results of benchmarking studies accordingly. We consider the simulators that show to be more promising by offering a combination of highly relevant features to biosecurity applications, including the ability to simulate realistic microbial communities, generate data from various sequencing platforms, incorporate sequencing errors, and provide gold standards for evaluating pipeline performance are CAMISIM, ART, and BEAR. Having these characteristics makes them particularly promising for generating HTS datasets that could aid in assessing the limit of detection and other diagnostic performance metrics of HTS in pathogen detection, which is critical for developing and implementing effective biosecurity measures.

**Table 2:** Key attributes of HTS dataset simulators that are useful for assessing HTS detection pipelines.

Simulator Name	Taxonomic Profiling (Metagenome)	Platform Errors	Genomic Variants	Sequencing Platform	Quality Score	Ref.	Latest Release Date [M/D/Y]
ART	It is designed for single genomes but can be adapted for metagenomics.	Yes, it simulates substitution and INDEL (insertion-deletion) errors.	Yes, with VarSim	Illumina	Yes	[32]	6/5/2016
BEAR	Yes, specifically designed for metagenomics.	Yes, emulates characteristics from real data.	Not specified, but designed to work with metagenomic datasets that inherently contain variations.	Ion Torrent, 454, Illumina	Yes	[42]	5/8/2020
CAMISIM	Yes, can model different microbial abundance profiles, multi-sample time series, and differential abundance studies.	Yes, offers flexibility in simulating various error profiles.	Yes, includes real and simulated strain-level diversity.	Illumina, PacBio, Oxford Nanopore	Yes	[34]	1/4/2022
CuReSim	No.	Yes, allows adjustments to error distribution along reads.	Yes, can introduce insertions, deletions, and substitutions at a controlled rate.	Ion Torrent	Yes	[35]	6/24/2015
FASTQSim	Does not allow profile input, but it has been used for metagenome simulations.	Yes, designed to be platform-independent and simulate various NGS datasets.	Yes.	Platform-independent	Yes	[33]	11/15/2016
Grinder	Yes, can simulate metagenomic data. User-defined profile or inferred from real HTS runs.	Yes, provides options for uniform, linear, and polynomial error models.	Not explicitly specified.	Sanger, 454, Illumina	Yes	[36]	11/27/2016
metaSPARSim	Yes, specifically designed for 16S rRNA gene sequencing data.	Yes, utilizes a Multivariate Hypergeometric distribution to model sequencing and simulate realistic sparsity and compositionality.	Not explicitly specified.	Not specified	Not specified	[39]	12/1/2020
MetaSim	Yes, explicitly designed for simulating metagenomic data.	Yes, supports user-defined parametric error models.	Not explicitly specified.	454, Illumina	No	[37]	10/8/2008
NanoSim	Yes, they added metagenomic simulation option.	Yes.	Not explicitly specified.	Nanopore	No	[41]	8/16/2024
NeSSM	Yes, designed for metagenomic sequencing simulation.	Yes, incorporates sequencing error models based on the distribution of errors at each base and coverage bias.	Not explicitly specified.	454, Illumina	Yes	[38]	8/18/2024
nfcore-ReadSimulator	Yes.	YES, from ART and capsim.	Not explicitly specified.	Illumina, PacBio		[43]	4/26/2024
ReadSim	Not explicitly specified.	Yes.	Not explicitly specified.	Nanopore, PacBio	Yes	[44]	12/1/2014
ReSeq	Yes.	Yes.	Yes.	Illumina, BGI	Yes	[40]	12/1/2020

## 4. Diagnostic Performance Metrics with Artificial HTS Datasets

Currently, there is no agreement on what tiers of diagnostic validation are required for HTS-based plant health diagnostics. The literature does not reflect a systematic way of validation where comparable results could aid in evaluating bioinformatic pipelines. However, prior literature has focused on a comprehensive validation approach encompassing key performance criteria tailored to the specific context of HTS technology. Rather than providing methodological procedures, generalized aspects have been suggested, adhering to established international standards such as ISO/IEC 9000:2015, ISO/IEC 17025:2017, and EPPO standards (e.g., PM 7/98, PM 7/147) for validation [45]. Such standards provide a framework for demonstrating that a test method consistently meets predefined requirements for its intended use. However, these standards have been merely designed for molecular assays, and HTS tests have not been added. Researchers have employed a range of diagnostic performance metrics to evaluate the efficacy of HTS tests. 

### 4.1. Analytical Sensitivity

Analytical sensitivity is a metric that reflects the ability of an HTS test to consistently detect a target pathogen at its lowest concentration. It is essentially the limit of detection (LoD) observed under ideal testing conditions [46]. Unlike traditional diagnostic tests, there is no simple formula that could be used for HTS tests. Determining analytical sensitivity for HTS tests requires establishing thresholds, including the minimum number of reads per sample and the determination of contamination levels [45]. Several factors can influence the analytical sensitivity of an HTS test. These factors include the number of reads generated from a sample, the level of contamination between samples, and the presence of co-infecting organisms.

When using bioinformatic pipelines to determine the presence/absence of pathogens in an HTS test, the analyte that we want to detect is pathogen reads, percentage of genome coverage, or depth of coverage. Determining the threshold has been a subjective matter; however, given the nature of HTS and the likelihood of having false positives due to the background noise found in most HTS runs, a Limit of Blank (LoB) should be calculated. The LoB is the highest concentration of an analyte, which is expected when running replicates of a sample without the analyte. The LoB is determined by calculating the mean result and standard deviation of replicate analyses of a blank sample [47].

The LoB is used to determine the limit of detection (LoD), which represents the lowest concentration of an analyte that can be reliably distinguished from the LoB [47]. This is important in HTS tests, where analytical sensitivity, represented by the LoD, is essential for distinguishing true positives from background noise. While the LoB helps establish a baseline for analytical signals in the absence of an analyte, it serves as a starting point for estimating the LoD rather than a direct measure of analytical sensitivity. The LoD is calculated by adding 1.645 times the standard deviation of a low-concentration sample to the LoB [47]. Once the LoD is established, it is confirmed by running the samples containing the LoD concentration and analyzing the observed values. The LoB is just one factor considered when determining and verifying the analytical sensitivity of HTS tests. For instance, a higher number of reads increases the probability of detecting a target, but it also raises the risk of detecting contamination if the bioinformatic pipeline thresholds are not well validated. Therefore, sequencing depth is a crucial metric when determining the analytical sensitivity of an HTS test. Other factors like duplication rate and host sequence abundance might also need consideration. Artificial HTS datasets can be beneficial in determining the analytical sensitivity of HTS-based plant pest diagnostic tests. Artificial datasets can supplement this gap since it is generally not feasible to have reference material encompassing every potential target and matrix combination for HTS tests. Examples of HTS simulators that can be used for this purpose include those that allow the user to modify the taxonomic profile, such as BEAR, CAMISIM, Grinder, metaSPARSim, MetaSim, NanoSIm, NESSM, nfcore-ReadSimulator, and ReSeq [34,36,37,38,39,40,41,42,43]. Rarefaction can be performed on real HTS runs to generate subsamples that mimic lower pathogen reads [48,49], but it is challenging to determine the original amount of pathogen reads, making it difficult to figure out the actual total read output. In this case, trial and error experiments may be necessary. Using HTS simulators that allow taxonomy modification can provide a more accurate representation of the pathogen.

### 4.2. Analytical Specificity

Analytical specificity, in its general sense, refers to the HTS test’s capability to detect all intended target pathogens (inclusivity) while avoiding the detection of unrelated pathogens (exclusivity) or host plant material (selectivity). This ensures the test is specifically targeting the desired organisms [46]. However, given the complexity of HTS tests for plant pest diagnostics and the complex interplay of factors, analytical specificity represents the ability of the HTS test to correctly identify the target organism at the desired taxonomic level without being misled by the presence of other organisms or genetic material in the sample. There is no specific metric or calculation for analytical specificity in HTS tests. However, the desired taxonomic resolution acts as a practical measure of analytical specificity. This means determining how effectively the HTS test can differentiate between organisms at the level required for its intended purpose—be it strain, species, genus, or family level identification. Artificial HTS datasets can play a significant role in evaluating the analytical specificity of HTS tests, particularly by addressing some of the limitations of relying solely on real-world samples, whose true composition is never fully known, making it challenging to determine if a negative result is truly negative or a false negative. In contrast to real datasets, artificial HTS datasets are entirely controlled in their composition. This means researchers know exactly which organisms and variants are present and at what frequencies. This precise knowledge is essential for evaluating whether an HTS test can accurately identify all the intended targets while excluding closely related organisms or common contaminants. By using artificial datasets, researchers can isolate and understand the impact of specific factors on taxonomic resolution. These factors might include sequencing depth, the presence of closely related organisms, or the choice of bioinformatic parameters. The knowledge gained from these controlled experiments can then be applied to improve the design of HTS tests, optimize bioinformatic analysis pipelines, and minimize the risk of false positives or negatives.

### 4.3. Diagnostic Sensitivity

The diagnostic sensitivity, often expressed as a percentage, reveals the proportion of truly positive samples accurately identified by the HTS test in an experiment utilizing known positive controls. It provides insight into the test’s ability to minimize false negatives. At this validation stage, the positive controls utilized should include the matrix where the infection occurs. However, it is very difficult to have reference material for every possible target and matrix combination due to the vast diversity of organisms detectable by HTS tests [45]. The calculation of diagnostic sensitivity relies heavily on several factors, including detection thresholds. Depending on the bioinformatic tool used to determine the presence or absence of the pathogen, when using tools like mapping to reference or read binning algorithms, a threshold must be selected. Selection of such a threshold should be performed with care because lowering the detection threshold, such as considering a single read as a positive detection, can increase diagnostic sensitivity. However, this approach can inflate the false positives, underscoring the importance of establishing appropriate thresholds. Additionally, the total number of reads generated, or sequencing depth, significantly impacts the ability to detect a target, particularly at low concentrations. A higher number of reads increases the likelihood of capturing target sequences, leading to higher sensitivity. Attempts have been made to generate thresholds for HTS tests; specifically, four genera of plant pathogens affecting citrus (a virus, two bacteria, and a viroid) have been used as a proof of concept where a quantitative discriminant analysis (QDA) was employed to establish a limit of detection (LoD) for each targeted pathogen. This LoD serves as a baseline score for determining positive diagnostic results. The LoD, representing the lowest detectable quantity of an analyte, is directly linked to the concept of analytical sensitivity. In the context of HTS and other diagnostic assays, thresholds are often used to define the boundaries between positive and negative results based on factors like the LoD. The use of a LoD calculated through QDA suggests that HTS test validation can employ a threshold-based approach. The calculated LoD could function as a practical threshold for interpreting the significance of pathogen signals in their analysis [31].

### 4.4. Diagnostic Specificity

Diagnostic specificity refers to the ability of a diagnostic test to correctly identify samples that do not have the target pathogen. In simpler terms, it measures how well a test avoids giving false positive results. A high diagnostic specificity indicates that the test is good at ruling out the presence of the target condition when it is truly absent. Evaluating diagnostic specificity is crucial during the development of any diagnostic assay, particularly for HTS applications in plant health diagnostics. This is because HTS methods have the potential to detect a wide range of organisms in a sample, making it crucial to differentiate true positives from false positives.

One significant challenge in calculating diagnostic specificity for HTS-based diagnostics is the potential for false positives arising from various sources, such as contamination or cross-reactions with similar organisms. This challenge is amplified by the fact that the complete range of organisms that an HTS test might detect is often unknown. Simulated HTS datasets are invaluable tools for addressing this challenge and accurately determining the diagnostic specificity of bioinformatic pipelines. The controlled composition of these datasets enables researchers to precisely evaluate the pipeline’s performance in identifying only the target organisms and avoiding misclassifications. Simulated datasets are important because they provide a known negative background, which is often difficult to define with real-world samples where the presence or absence of all potential organisms is uncertain. By spiking known concentrations of target organisms, researchers can assess the pipeline’s ability to correctly identify these targets while ignoring unrelated sequences, thus providing a direct measure of diagnostic specificity. Mock datasets can be designed to include challenging scenarios, such as low virus titers, the presence of closely related organisms, or sequencing errors, to test the robustness of the pipeline’s specificity under these conditions.

### 4.5. Precision

Precision in HTS diagnostics, which encompasses repeatability, intermediate precision, and reproducibility, ensures consistent outcomes across various operators, equipment, and laboratories. Simulated HTS datasets are valuable tools for evaluating precision because they allow researchers to control variables like pathogen prevalence and sequencing error rates. By generating datasets with predefined characteristics and analyzing them using different operators, equipment, or laboratories, researchers can assess the variability in results. Analyzing these variations provides insights into the robustness of the HTS test and helps establish the reliability of results across different testing environments.

### 4.6. Robustness

Assay robustness refers to the ability to maintain precision when experiencing minor variations in protocol or environmental conditions. Although no specific formula exists for calculating robustness, it involves identifying potential sources of variation, such as changes in reagent concentrations, incubation times, thermocycling conditions, fluctuations in laboratory temperature, humidity, equipment calibration, and differences in operator expertise. For the HTS test, deliberate variations should be systematically introduced within a defined range around the standard protocol, and well-characterized positive and negative controls should be included to assess the impact on assay performance. The results are analyzed to evaluate the effect of variations on key performance metrics like sensitivity, specificity, and the limit of detection, and to define the range of variation that maintains acceptable assay performance. Any protocol deviations and their impacts should be clearly documented. Verification studies are emphasized to assess the impact of planned deviations from a validated method, ensuring the test’s robustness to modifications. In the context of HTS tests used for plant health diagnostics, simulated HTS datasets offer distinct advantages over real datasets, such as controlled composition, ground truth knowledge, reproducibility, and the ability to address specific diagnostic challenges. Simulated datasets allow complete control over variables like pathogen concentration, mutation frequencies, and contaminant presence, aiding in targeted benchmarking and validation of specific bioinformatic pipeline features. Researchers have full knowledge of the ‘true’ composition of simulated datasets, eliminating the uncertainty associated with real-world samples, thus facilitating accurate performance evaluation and unbiased comparison of different bioinformatic pipelines. Moreover, simulated datasets enable consistent, reproducible benchmarking conditions, unlike real datasets subject to natural variations and potential batch effects. Researchers can design simulated datasets to tackle particular diagnostic challenges, such as detecting low-abundance viruses, identifying novel strains, or disentangling complex infections, aiding in developing and evaluating pipelines tailored to these challenges.

## 5. Conclusions

In conclusion, simulated HTS datasets play a crucial role in evaluating and validating the diagnostic performance of HTS tests for plant pathogen detection. These datasets offer a controlled and reproducible environment for assessing key performance metrics such as analytical sensitivity, analytical specificity, and precision. By generating datasets with predefined characteristics, such as pathogen prevalence, genome complexity, and sequencing error rates, we can systematically evaluate the impact of these variables on the ability of HTS tests to accurately detect and identify plant pathogens. The availability of a wide array of simulators makes it difficult to select the simulator that works best for determining the diagnostic performance characteristic metrics of HTS tests. In this article, we aimed to bring awareness of the complexities of evaluating HTS tests and that selecting the appropriate simulator or having standardized HTS reference controls is a potential solution to the lack of consensus on how to evaluate HTS tests for agricultural diagnostic purposes. This controlled approach helps researchers establish reliable detection thresholds, minimize false-positive and false-negative results, and ensure the robustness of HTS tests across different laboratories and testing conditions. The insights gained from analyzing simulated HTS datasets contribute significantly to the development and implementation of accurate and dependable HTS-based diagnostic tools for plant health management.

## Figures and Tables

**Table 1:** Overview of assay performance metrics, aims, variables, and appropriate HTS dataset types.

Performance Metric	Aim	Variables	HTS Dataset Type
Analytical sensitivity	Determine the lowest concentration of the target that is consistently detectable.	Quantitative: Limit of Detection (LoD) calculated as the mean of replicate tests used to calculate the Limit of Blank (LoB) + 1.645 standard deviations of a low-concentration sample. Qualitative: the lowest concentration is consistently detected as positive in repeated testing.	Real: serially diluted samples. Simulated: datasets with varying target concentrations.
Analytical specificity	Ensure the assay accurately detects all target variants (inclusivity) while excluding non-targets (exclusivity).	Inclusivity: percentage of target variants correctly identified. Exclusivity: percentage of non-target samples correctly identified as negative. Selectivity: ability to detect target in the presence of background matrix.	Real: panels of target and non-target samples, including closely related species and environmental samples. Simulated: datasets containing a mix of target and non-target sequences with controlled variations.
Diagnostic sensitivity	Evaluate the assay’s ability to correctly identify true positive samples.	Percentage of known positive samples correctly identified by the test.	Real: panels of samples with confirmed presence or absence of the target. Simulated: not suggested.
Diagnostic specificity	Assess the assay’s ability to correctly identify true negative samples.	Percentage of known negative samples correctly identified.	Real: panels of samples with confirmed absence of the target. Simulated: they are needed because they provide a known negative background. The intentional inclusion of closely related organisms to the target is suggested (see precision).
Precision	Precision refers to the closeness of agreement between independent test results obtained under specified conditions.	Repeatability: this involves assessing the variation when the same operator conducts the assay on the same sample multiple times. Intermediate precision: agreement between results from multiple operators or instruments within a lab. Reproducibility: evaluate the variation in results when the assay is performed in different laboratories and by different operators.	Real: replicate testing of the same samples. Simulated: datasets containing a mix of target and non-target sequences with controlled variations and concentrations.
Robustness	Ability to maintain its precision despite variations in factors. It evaluates the assay’s performance under variable conditions.	The ability of the test to maintain precision when subjected to variations. Most variations for bioinformatic pipelines come from the operator. These are deliberate variations. In this case, variations in the pipeline could be intentionally introduced, i.e., read filtering, library size (rarefaction), and slight variation in pathogen reads, incorporating closely related organisms, etc. Typically assessed through ring tests involving multiple laboratories.	Real: testing under various conditions and with minor protocol deviations. The variations are more limited. Simulated: variations that include pathogen read abundance and library size read filtering, among others, can be performed without restrictions.

## Data Availability

Not applicable.
